# Heme oxygenase-1: emerging target of cancer therapy

**DOI:** 10.1186/s12929-015-0128-0

**Published:** 2015-03-21

**Authors:** Lee-Young Chau

**Affiliations:** Institute of Biomedical Sciences, Academia Sinica, Taipei, 115 Taiwan

**Keywords:** Heme oxygenase-1, Oxidative stress, Inflammation, Tumorigenesis

## Abstract

Heme oxygenase-1 (HO-1) is a rate-limiting enzyme catalyzing oxidative degradation of cellular heme to liberate free iron, carbon monoxide (CO) and biliverdin in mammalian cells. In addition to its primary role in heme catabolism, HO-1 exhibits anti-oxidative and anti-inflammatory functions via the actions of biliverdin and CO, respectively. HO-1 is highly induced in various disease states, including cancer. Several lines of evidence have supported the implication of HO-1 in carcinogenesis and tumor progression. HO-1 deficiency in normal cells enhances DNA damage and carcinogenesis. Nevertheless, HO-1 overexpression in cancer cells promotes proliferation and survival. Moreover, HO-1 induces angiogenesis through modulating expression of angiogenic factors. Although HO-1 is an endoplasmic reticulum resident protein, HO-1 nuclear localization is evident in tumor cells of cancer tissues. It has been shown that HO-1 is susceptible to proteolytic cleavage and translocates to nucleus to facilitate tumor growth and invasion independent of its enzymatic activity. HO-1 also impacts cancer progression through modulating tumor microenvironment. This review summarizes the current understanding of the protumorigenic role of HO-1 and its potential as a molecular target for cancer therapy.

## Review

Heme oxygenase-1 (HO-1) was initially identified as a liver microsomal protein with activity to degrade heme to bilirubin four decades ago [1]. The first HO-1 cDNA was cloned from rat spleen in 1985 [2], and the amino acid sequence revealed a hydrophobic segment located at the carboxyl terminus, which is required for its anchoring to endoplasmic reticulum (ER) with type II transmembrane topology. HO-1 is highly expressed in the organs responsible for degrading senescent red blood cells, including spleen, reticuloendothelial cells of the liver and bone marrow, and HO-1 in macrophages is involved in the recycling of hemoglobin-heme [1]. Although the basal HO-1 expression level in tissues not directly responsible for hemoglobin metabolism is low, HO-1 is highly induced in cells upon exposure to many agents promoting cellular stresses, including heavy metals, endotoxin, cytokines, heme, hypoxia, nitric oxide, and UV irradiation [2-6]. These findings indicate that HO-1 is not only involved in normal physiology but also has a role in pathophysiological states.

The research on HO-1 was rapidly extended in late 1990’s. It has been shown that HO-1 knockout mice develop anemia associated with hepatic and renal iron overload, which leads to oxidative tissue injury and chronic inflammation [7]. Moreover, these mice are more susceptible to ischemic and reperfusion injury [8,9]. A human case of HO-1 deficiency was also reported [10]. This patient suffered from growth retardation with persistent hemolytic anemia and abnormal coagulation/fibrinolysis system associated with persistent vascular injury. These observations provide strong evidence to support the implication of HO-1 in systemic iron homeostasis and stress response. Along with the genetic studies, increasing evidence has accumulated to show that CO and biliverdin, the byproducts derived from heme degradation by HO-1, possess potent anti-inflammatory and antioxidant activities, respectively [11,12]. These findings provoke substantial interest in the cytoprotective functions of HO-1 and its therapeutic potential in treating various disease states associated with inflammation and oxidative stress, such as cardiovascular and pulmonary diseases [13]. Considering that both oxidative stress and inflammation are implicated in tumorigenesis, the role of HO-1 in cancer has also received considerable attention in recent years.

### Association between HO-1 gene polymorphism and cancer

Genetic variation is one of the important factors contributing to cancer susceptibility. A (GT)n repeat polymorphism present in the proximal promoter of human HO-1 gene has been shown to influence the transcriptional activation of HO-1 gene [14]. The shorter (GT) repeats is associated with higher transcriptional activity of HO-1 gene in human cells. Several genetic studies have been carried out to assess the association between HO-1 gene polymorphism and the risk of cancer in humans [15-23]. It has been shown that subjects carrying the shorter (GT) repeats have lower risk in oral squamous cell carcinoma [15], lung adenocarcinoma [16], gastric adenocarcinoma [18], breast cancer [19], esophageal squamous cell carcinoma [21], and malignant mesothelioma [23]. However, some reported the higher risk for melanoma [17], gastric cancer [20], and pancreatic cancer [22] in subjects carrying the shorter repeats. The inconsistency is likely caused in part by the differences in the subject ethnicity, sample sizes, disease stages, and cancer types for studies [24]. It is apparent that further studies with large homogeneous patient populations will be needed to validate the association between HO-1 gene polymorphism and human cancer.

### Association between HO-1 expression and cancer progression

HO-1 overexpression is commonly seen in human cancers, including prostate [25], renal [26], gastric [27], colon [28], lung [29], thyroid [30], bladder [31], breast [32], oral [33], and glioma [34] cancers. Moreover, the expression level is positively correlated with the disease stage and poor prognosis in patients. Notably, there were studies showing that HO-1 was detected not only in cytoplasm but also in nucleus of tumor cells in prostate [35,36], lung [29,37], and oral [38] cancer tissues. The extent of HO-1 nuclear localization was associated with disease progression and poor prognosis in patients with prostate cancer [36] and oral carcinoma [38]. In addition to the expression in tumor cells, the positive HO-1 immunoreactivity was also detected in stromal compartment, particularly the tumor-associated macrophages [39-41] of cancer tissues, suggesting that HO-1 may impact cancer progression through modulating tumor microenvironment.

### Regulation of HO-1 expression in cancer cells

Cancer cells exhibit elevated oxidative stress due to their high metabolic rate. Moreover, they are surrounded by a complex microenvironment and significantly influenced by their interplays with the stromal components, especially the infiltrating inflammatory cells [42]. It is envisioned that the oxidative stress and stimulations by various growth factors and cytokines released from stromal cells are capable of inducing HO-1 gene transcription in tumor cells through activation of various signaling pathways and transcriptional factors, including Nrf2, NF-κB, AP2 and others [43]. Hypoxia has also been shown to induce HO-1 expression [6].

Furthermore, HO-1 gene expression is upregulated by oncogenes, such as Kaposi sarcoma-herpes virus [44] and BCR/ABL kinase [45]. In addition to the regulation at transcriptional level, HO-1 expression is subjected to posttranscriptional regulation. It has been shown that regulation of HO-1 by mir378 is implicated in lung carcinoma growth and metastasis [46]. Downregulation of HO-1 by mir200c enhances the sensitivity of renal carcinoma cells to chemotoxic agents [47]. Moreover, HO-protein is turnovered by ubiquitin-proteasome system [48]. Our group recently demonstrated that HO-1 is a physiological substrate of TRC8, which is an ER-resident E3 ligase associated with hereditary renal cell carcinoma and thyroid cancer [49]. It was shown that the tumor suppressive effect of TRC8 is mediated at least in part via targeting HO-1 for ubiquitination and degradation in cancer cells.

### Paradoxical roles of HO-1 in tumorigenesis

Tumorigenesis is a multistep process in which the accumulation of several genomic mutations is required to initiate the transformation of normal cells to become cancer cells. DNA damage caused by the reactive oxygen species (ROS) is a major source of mutation. HO-1 downregulation leads to the increase of ROS and DNA damage in cells [49]. Furthermore, CO improves cell survival post irradiation or genotoxin treatment by inducing DNA repair [50]. Therefore, increase in HO-1 expression prevents DNA damage and the initiation of carcinogenesis in normal cells. However, at late phase of tumorigenesis, HO-1 overexpression promotes cancer cell proliferation and invasiveness [45,49,51-53]. HO-1 protects cancer cells from apoptosis induced by chemotoxic agents or irradiation, suggesting its involvement in therapeutic resistance [54-61]. A recent study showed that CO contributes to the resistance of cancer cells to oxidative stress and chemotoxic agents by inhibiting the heme-containing cystathionine β-synthase, which causes reduced PFKFB3 methylation and shift of glucose metabolism to pentose phosphate pathway, resulting in subsequent increase of NADPH to replenish reduced glutathione [62].

Paradoxically, another study in prostate cancer demonstrated that CO inhibits tumor growth and increases sensitivity to chemotherapy by enhancing metabolic exhaustion [36]. The cause behind the opposite effects of CO observed in these studies is not yet clear. Nevertheless, HO-1 augments angiogenesis in tumor by inducing the expression of angiogenic factors, such as vascular endothelial growth factor (VEGF) [63-65]. Recently, increasing evidence has demonstrated the involvement of Nrf2-mediated transcriptional activation of antioxidant genes in promoting cell transformation and tumorigenesis [66,67]. Mutations in Nrf2 and its inhibitor, KEAP1, have recently been identified in human cancers [68]. As HO-1 is one of the target genes regulated by Nrf2, it is apparent that HO-1-mediated antioxidant response contributes at least in part to the tumorigenic process promoted by Nrf2 activation. Targeting HO-1 has been shown to be an effective approach for hormone-refractory prostate cancer [69] and overcome imatinib resistance in chronic myeloid leukemia [70]. It also increases the sensitivity of hepatoma, urothelial and pancreatic cancers to chemotherapy [55,60,63,71]. Furthermore, HO-1 inhibition is synthetic lethal in fumarate-hydrotase deficient cells associated with hereditary leiomyomatosis and renal-cell cancer [72].

### Protumorigenic function of nuclear HO-1

Although HO-1 is an ER-anchored protein, there were reports showing HO-1 localization in other subcellular compartments [73-75]. HO-1 nuclear localization has been seen in fetal lung cells exposed to hyperoxia [76], and in brown adipocytes and astroglial cells during differentiation [77,78]. HO-1 nuclear localization was also evident in the cancer cells of prostate, lung, and oral cancer tissues, and associated with tumor progression [29,35,36,38]. However, the pathophysiological significance of HO-1 nuclear localization and the mechanism involved are not yet fully explored. Early studies have shown that HO-1 is sensitive to proteolytic cleavage [79]. A recent study demonstrated that HO-1 undergoes proteolytic cleavage, which results in the release of a soluble HO-1 with truncation of its C-terminal transmembrane segment from ER membrane and subsequent translocation to nucleus under some stress conditions in vitro [80]. More recently, studies from our group and others have shown that HO-1 is susceptible to intramembrane cleavage mediated by the ER-associated signal peptide peptidase (SPP) [37,81]. We further demonstrated that SPP is highly expressed in lung cancer cells, and correlates with HO-1 nuclear localization in the same lung cancer tissues [37]. Interestingly, nuclear HO-1 promotes tumor growth and invasion independent of its enzymatic activity [37]. The nuclear HO-1 translocation has also been shown to be implicated in imatinib resistance in chronic myeloid leukemia cells [82]. These findings add a new dimension to HO-1-mediated protumorigenic effects. It has been shown that HO-1 nuclear translocation confers protection against oxidative stress in yeast and mammalian cells through activating oxidant responsive transcriptional factors and upregulation of antioxidant genes. [80,83] Since HO-1 does not contain DNA binding domain, whether it can impact the transcription of genes related to cancer progression through interaction with transcriptional factors or other nuclear proteins deserves further investigation.

### Impacts of hematopoietic HO-1 on cancer

The immune/inflammatory cells recruited to tumor microenviroment have profound effects on cancer progression by modulating inflammatory response and anti-tumor immunity [42]. HO-1 has been shown to modulate the immune regulatory functions of myeloid cells by suppressing the expression of proinflammatory cytokines, such as tumor necrosis factor-α, but promoting the expression of immunosuppressivecytokine, interleukin-10 (IL-10) [12]. HO-1 promotes inflammation-associated angiogenesis through up-regulating VEGF expression in macrophages [84]. Furthermore, HO-1 expression in myeloid-derived suppressor cells participates in the suppression of alloreactive T cells [85]. Although HO-1 expression in the stromal macrophages has been seen in the cancer tissues [39-41], the impact of HO-1 expression in myeloid cells on cancer progression is less explored. A recent study by Arnold et al. demonstrated that HO-1 expression mediates the immune suppressive function of a stromal macrophage subpopulation expressing fibroblast activation protein-α [86].

By performing the syngeneic tumor graft experiments with wild type and HO-1 ^+/−^ mice, we recently demonstrated that the host HO-1 expression did not affect the growth of primary tumor, but significantly enhanced lung metastasis [87]. The involvement of hematopoietic HO-1 in this process was further demonstrated by the bone marrow transplantation experiment. Mechanistically, we found that HO-1 enhances the chmoattractant-induced migration response of myeloid cells, and therefore facilitates the recruitment of myeloid cells to the pulmonary premetastatic niche and the metastatic loci. Moreover, myeloid HO-1-induced expressions of VEGF and IL-10 promoted tumor cell extravasation and STAT3 activation, which are crucial for the survival and successful colonization of tumor cells in metastatic sites.

In addition to the role in innate immunity, HO-1 also participates in the adaptive immune response in tumor microenvironment. There was a study showing that HO-1-specific CD8^+^ regulatory T cells with immunosuppressive activity is present in the peripheral blood and tumor tissues of patients [88]. Collectively, these findings support that HO-1 can impact cancer progression through modulating tumor microenvironment.

## Conclusion

**Figure 1:**
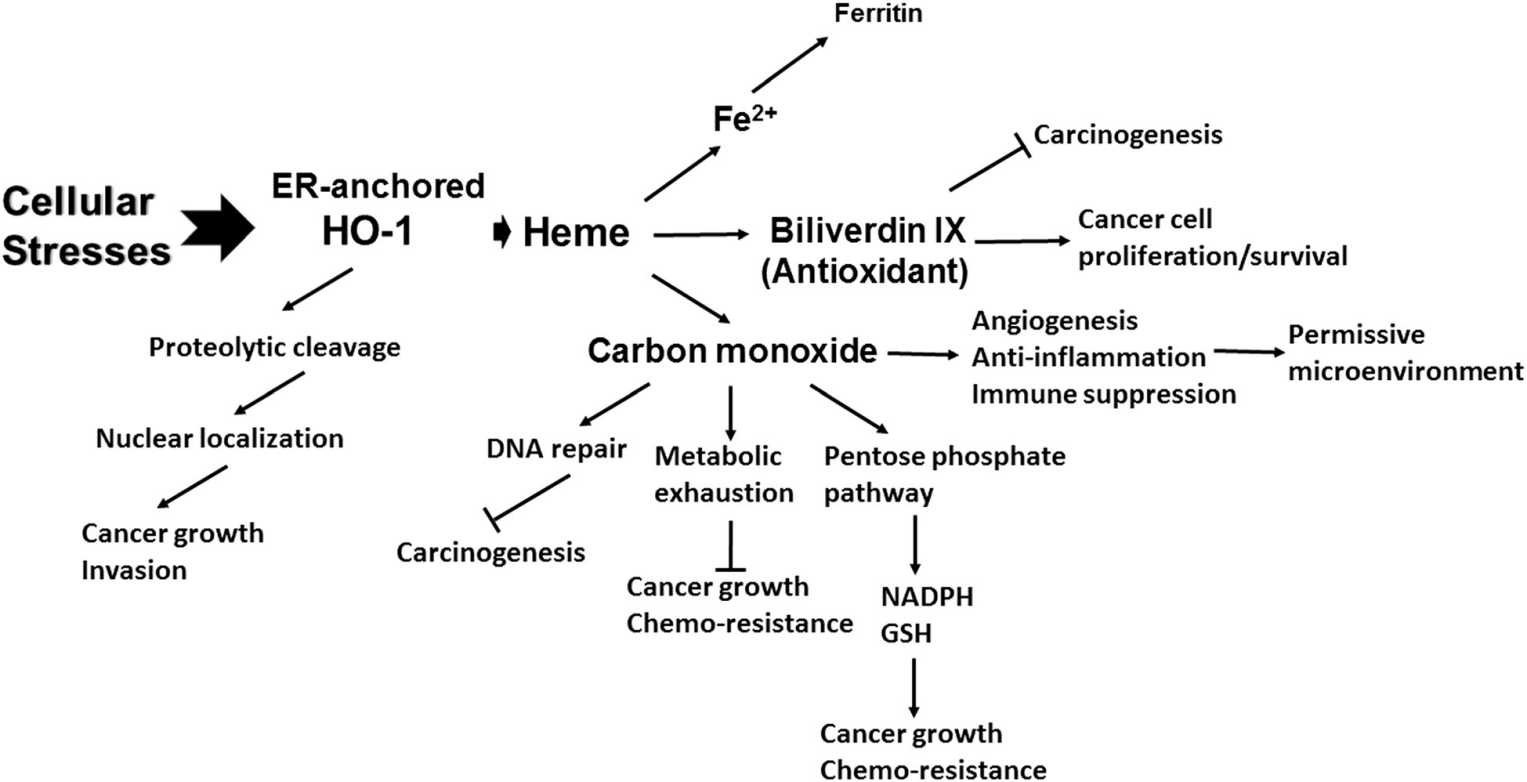
**Multifaceted roles of HO-1 in cancer.** HO-1 induction under various cellular stresses impacts tumorigenesis through multiple pathways. CO and biliverdin, the byproducts derived from heme degradation by HO-1 reaction, protect normal cells from transformation in the early phase of tumorigenesis, whereas promote the growth and survival of tumor cells in the late phase of cancer development. Nuclear translocation of HO-1 from ER can affect cancer progression independent of its enzymatic activity. Moreover, HO-1 expression in stromal compartments influences the establishment of cancer permissive microenvironment.

As illustrated in Figure 1, HO-1 can influence cancer development through multiple pathways. HO-1 confers protection in early carcinogenesis, but it promotes cancer cell survival, growth and metastasis in the later process. The paradoxical roles of HO-1 in different phases of tumorigenesis may provide partial explanation for the discrepant findings in the genetic association studies. Beyond its effect on tumor cells, HO-1 can impact cancer progression through modulating tumor microenvironment. Myeloid HO-1 expression promotes the recruitment of immune/inflammatory cells and their immunosuppressive and proangiogenic capacities facilitate cancer cell growth and metastasis. Although considerable protumorigenic effects of HO-1 are mediated by its reaction byproducts, the discovery that HO-1 translocates to nuclear to enhance tumor cell proliferation and invasion via a mechanism independent of its enzymatic activity increases the complexity of HO-1-targeted therapy. Nevertheless, accumulative evidence has demonstrated that HO-1 inhibition using specific gene knockdown approach or metalloprotoporphyrin competitive inhibitor, such as zinc protoporphyrin IX, to block heme binding significantly enhances the sensitivity of cancer cells to chemotherapy or irradiation and suppresses cancer metastasis in experimental animals [55,60,61,63,70.71]. Recently, several imidazole-based non-porphyrin HO-1 inhibitors were developed [89]. These compounds exhibit higher selectivity toward HO-1 without affecting other heme-containing proteins. Moreover, their potent anti-tumor activities have been shown in vitro and in vivo, supporting their therapeutic applications [69,89,90]. Collectively, these findings support the possibility of targeting HO-1 to improve cancer immunotherapy and prevent metastasis, which is the major cause of cancer-associated death.

## Abbreviations

CO: Carbon monoxide
ER: Endoplasmic reticulum
HO-1: Heme oxygenase-1
IL-10: Interleukin-10
ROS: Reactive oxygen species
VEGF: Vascular endothelial growth factor
